# Apparent diffusion coefficient metrics for treatment response assessment in soft tissue sarcoma: Reproducibility and prognostic value of whole-tumor and sub-regional analysis

**DOI:** 10.1007/s00256-026-05318-9

**Published:** 2026-07-30

**Authors:** Iris van der Loo, Valentina Valentini, Kalina Chupetlovska, Stephan Ursprung, Petra J. van Houdt, Annemarie Bruining, Regina G. H. Beets-Tan, Alex L. T. Imholz, Andrea Delli Pizzi, Stefano Trebeschi

**Affiliations:** 1 Department of Radiology, The Netherlands Cancer Institute, Amsterdam, The Netherlands; 2 Maastricht University, Maastricht, The Netherlands; 3 Research Office, Deventer Ziekenhuis, Deventer, The Netherlands; 4 Department of Radiology, Santissima Annunziata Hospital, University “G. d’Annunzio”, Chieti, Italy; 5 Department of Radiation Oncology, The Netherlands Cancer Institute, Amsterdam, The Netherlands; 6 The Netherlands Cancer Institute, Amsterdam, The Netherlands; 7 Faculty of Health Sciences, University of Southern Denmark, Odense, Denmark; 8 Maastricht Radiation Oncology, Maastricht, The Netherlands; 9 Department of Oncology, Deventer Ziekenhuis, Deventer, The Netherlands; 10 Department of Innovative Technologies in Medicine and Dentistry, University “G. d’Annunzio”, Chieti, Italy; 11 ITAB Institute for Advanced Biomedical Technologies, University “G. d’Annunzio”, Chieti, Italy

**Keywords:** Diffusion-weighted imaging, Apparent diffusion coefficient, Soft tissue sarcoma, Response assessment, Reproducibility

## Abstract

**Objective:**

To evaluate the inter-observer reproducibility and predictive value of apparent diffusion coefficient (ADC) metrics derived from diffusion-weighted MRI for assessing histopathologic response to neoadjuvant radiotherapy in soft tissue sarcoma (STS).

**Materials and methods:**

This retrospective, single-institution cohort study included 23 patients with histologically proven STS treated with neoadjuvant radiotherapy. Patients underwent 1.5 T or 3.0 T MRI, including diffusion-weighted sequences (b = 200, 800 s/mm^2^). Three independent radiologists measured mean tumor ADC, high-ADC, and low-ADC regions at baseline and post-radiotherapy. Reproducibility was assessed using the Intraclass Correlation Coefficient (ICC). Response prediction and clinicopathological associations were evaluated using Mann–Whitney U tests, logistic regression, and Spearman rank correlations.

**Results:**

Single-timepoint ADC measurements demonstrated high inter-observer reproducibility, particularly for mean tumor ADC (ICC = 0.91–0.95). Reproducibility for ADC changes over time was lower; agreement for mean tumor ADC (ICC = 0.79) outperformed subregional values (ICC = 0.06–0.63). Post-treatment mean tumor ADC was significantly elevated in responders (median 2.39 × 10⁻^3^ mm^2^/s) versus non-responders (1.50 × 10⁻^3^ mm^2^/s, *p* = 0.02). High follow-up ADC values (mean and high-ADC subregions) were the strongest predictors of pathological response (OR = 20.3–33.1, all *p* < 0.05). Follow-up ADC values inversely correlated with the percentage of viable tumor cells (*p* < 0.05).

**Conclusion:**

Post-treatment ADC measurements were highly reproducible and best predicted pathological response in our cohort. Longitudinal changes were less robust and require further research into the effect of standardized DWI protocols and more reliable techniques for sub-regional analysis.

**Supplementary information:**

The online version contains supplementary material available at https://doi.org/10.1007/s00256-026-05318-9.

## Introduction

Accurate and reproducible response assessment is critical for novel drug testing, yet remains particularly challenging in soft tissue sarcomas (STS). The size-based Response Evaluation Criteria in Solid Tumors (RECIST) have proven inadequate, as STS may paradoxically increase in size after effective neoadjuvant radiotherapy (nRT) due to edema, necrosis, or hemorrhage. To address this, the MR-adapted Choi criteria were introduced based on the original CT-based standards for gastrointestinal stromal tumors, substituting density for signal intensity [1, 2]. However, this method is limited by tumor heterogeneity [3–5] and considerable variability [6]. Pilot data showed full concordance in only 68% of RECIST cases and 42% for Choi. These rates dropped further when different radiologists performed baseline and follow-up measurements [6].

This variability also complicates clinical trials. Evaluating new therapies requires assessing efficacy through large phase III trials or meta-analyses of multiple (randomized) phase II trials to average out measurement error and estimate true treatment effect. However, STS are rare (~ 5 cases per 100,000) [7] and histologically diverse [7, 8]. As a result, large clinical trials are often not feasible, making reliable response assessment for small trials and real-world data an unmet clinical need.

New criteria should quantify treatment effect leveraging estimates of viable tumor, necrosis and hyalinization/fibrosis, before and after treatment. Literature shows that necrosis rates of < 90% correlate with higher recurrence [9], while pathologic complete response (pCR) and therapy-induced hyalinization and fibrosis improve survival [10, 11]. Since definitive pathology is only available post-resection, a radiological surrogate is needed to monitor changes during therapy.

Diffusion-weighted imaging (DWI) is a promising non-invasive technique. It is based on the biophysical difference in water mobility between viable tumor and necrotic or fibrotic tissue [12, 13]. In viable tumor tissue, high cellular density and intact membranes result in restricted diffusion [14]. Conversely, cell death and membrane disruption reduce this restriction. Consequently, quantitative analysis through Apparent Diffusion Coefficient (ADC) should enable objective evaluation of tumor viability [13, 15–17]. Although the European Society of Musculoskeletal Radiology [18] now recommends DWI, its clinical reliability requires further validation.

Previous studies on DWI in STS faced methodological limitations, including a lack of longitudinal data [19], absence of correlation with histopathology [20] or limited reader pools (maximum of two) [19–24]. This leaves the inter-observer variability of these metrics largely unestablished. To address these gaps, this exploratory study evaluates ADC as a non-invasive biomarker for treatment response. Specifically, we assess the predictive value and reproducibility of various ADC measurement techniques across multiple radiologists, using pathological response as the reference standard.

## Materials and methods

### Patient cohort

We collected a retrospective, single-institution cohort of patients with pathologically confirmed intermediate or high-grade STS between January 2010 and January 2025 at the Netherlands Cancer Institute. The study was approved by the local Institutional Review Board of the Netherlands Cancer Institute (IRB d22-347), and written informed consent was waived. All patients underwent MRI before and at the end of nRT. Baseline characteristics, including age, sex, histologic subtype and tumor location were collected. Surgical resection of the tumor was performed following completion of nRT.

### Imaging protocol

All included MRIs were acquired on 1.5 T or 3.0 T systems with coils adapted to tumor location and patient body. Scans had to include at least two orthogonal planes with T2-weighted (T2w), T1-weighted post-contrast fat-suppressed (T1w fatsat + C) and DWI. Only DWI scans meeting minimum image quality (slice thickness ≤ 8 mm, pixel spacing ≤ 2 mm) and sequence criteria (DWI with b = 200 and b = 800 s/mm^2^) were analyzed. ADC maps were generated using OSIPI Taskforce 2.4 (*generate_ADC_standalone*)^24^. To ensure compatibility between scans voxel-wise mono-exponential fitting (log-linear regression) was performed on b = 200 and b = 800 s/mm^2^ data, excluding b = 0 to minimize perfusion and T2 shine-through effects. ADC values are reported in × 10⁻^3^ mm^2^/s.

### Images analysis workflow

We recruited three radiologists, blinded to pathological outcomes and prior radiology reports. Image analysis was conducted using an in-house developed extension for 3D Slicer (v5.2.2 +). For each patient, the radiologists were first presented with the baseline scan and subsequently with the baseline plus follow-up scans, resembling the sequence of image review in clinical routine. ADC maps, DWI, T1w fatsat + C, and T2w images were displayed simultaneously.

Scan quality was assessed using a modified version of the Radiological Image Quality Score (RI-QUAL) [25], adapted to diffusion-weighted imaging of soft-tissue sarcomas. The score is based on a four-tier scale from A (optimal) to D (non-diagnostic) For scans rated “D”, radiologists provided a brief explanation of the limiting factor.

Three ROIs were defined to account for the inherent heterogeneity of STS: the total tumor volume (TTV), and the most hyper- and hypointense tumor regions. While TTV provides an aggregate assessment of the lesion, hypointense regions strongly correlate with areas of dense cellularity and tumor aggressiveness, whereas hyperintense regions with low cellularity, such as cystic, myxoid, or necrotic areas are critical for evaluating treatment response [26–28]. In addition to ADC, whole-tumor volume was derived from the TTV segmentation at both timepoints and averaged across the three readers to assess treatment-related size change.

To speed up the contouring process, initial TTV segmentations were generated on the T1w fatsat + C sequences using an in-house nnU-Net [29]. A detailed description of this algorithm falls outside the scope of this work, as all AI outputs were strictly used for initialization and subsequently manually refined. The preliminary masks were transferred to the ADC maps using an identity transform with nearest-neighbor interpolation. Because this automated spatial alignment assumes the patient did not move, all masks needed to be manually reviewed and adjusted to correct for inter-scan motion and model imprecision.

**Fig. 1:**
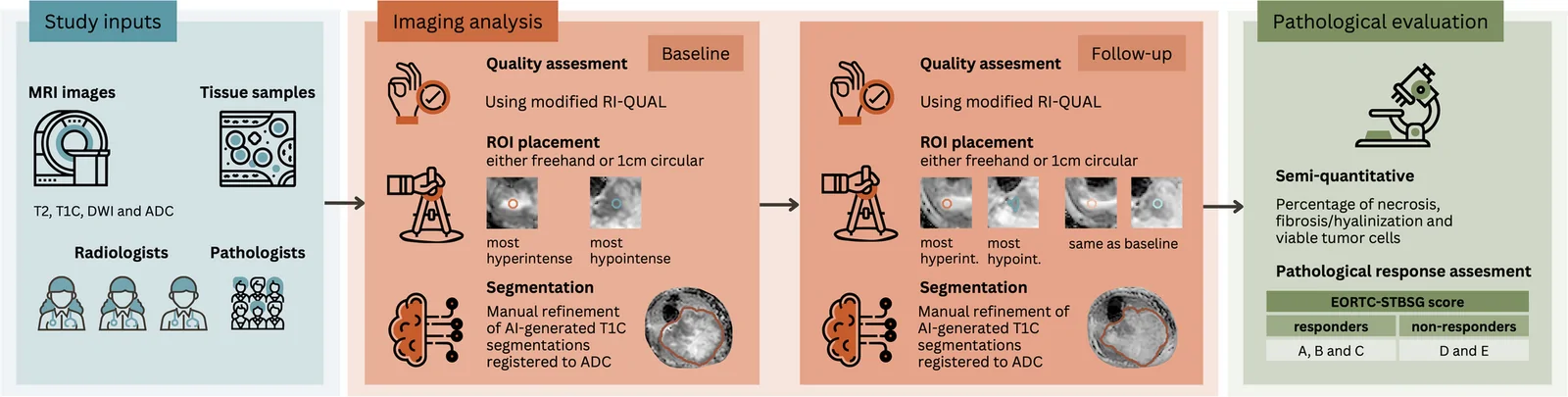
Graphical overview of the study design and image analysis workflow**.** Radiologists reviewed scans using a structured protocol involving quality scoring, specific region of interest selection strategies and semi-automated segmentation. These findings were compared against the pathological response assessment

Focal hyper- and hypointense ROIs were manually delineated within visually homogeneous areas using a standard 1-cm circular ROI, or a freehand ROI when anatomically necessary. Rather than restricting ROI selection to a fixed slice, readers assessed the entire tumor volume to identify the region with the most extreme ADC value and placed the ROI within a well-defined, visually homogeneous portion of that region. Final ROI placement was left to reader discretion. This process was repeated for follow-up scans, with the addition of 'Same as Baseline' (SaB) ROIs placed in anatomically corresponding regions as their corresponding baseline ROIs to enable longitudinal comparison (Fig. 1).

### Histopathological evaluation

Pathological analysis of surgical specimens was conducted by dedicated sarcoma pathologists. Representative tissue sections were sampled from multiple macroscopic tumor regions to ensure a comprehensive assessment of overall tumor response. A semi-quantitative score estimated the percentage of necrosis, fibrosis/hyalinization (“fibrosis”), cavity/hematoma, myxoid change, mature tissue and viable tumor cells (defined as those retaining staining characteristics). Pathologic response to neoadjuvant treatment was assessed using the EORTC–STBSG score, which classifies resection specimens based on the proportion of viable tumor cells: Grade A (no viable cells), Grade B (< 1%), Grade C (1–9%), Grade D (10–49%), and Grade E (≥ 50%) [30]. For this study, responders were defined as patients with < 10% viable tumor cells, encompassing both complete (pCR, Grade A) and near-complete (near-pCR, Grades B–C) response, following the EORTC-STBSG guidelines [30].

### Statistical analysis

All statistical analyses were performed using R software (version 4.5.0). Continuous variables were reported as mean ± standard deviation (SD) or median and interquartile range (IQR) and categorical variables as frequencies and percentages. Comparisons were performed using Student’s t-test or Mann–Whitney U test, with *p*-values adjusted for multiple testing using the Benjamini–Hochberg method. For treatment-related changes over time ΔADC was calculated as:

$$\Delta ADC = (AD{C}_{follow-up}-AD{C}_{baseline})/AD{C}_{baseline}$$

Inter-reader repeatability was assessed using the intraclass correlation coefficient (ICC) for continuous variables and Fleiss’ kappa for quality scores (on the original 4-point scale and dichotomized as A + B vs. C + D). Consensus ADC values were derived as the mean of the three readers. Variables showing a statistically significant difference between responders and non-responders were further analyzed using univariable logistic regression. A two-sided *p*-value < 0.05 was considered statistically significant.

## Results

### Patient characteristics

A total of 23 patients were included in the study. Two patients did not have final pathological reports available at our institute; they were therefore excluded from the pathological response analysis but remained included for the inter-observer variability assessment. Among the 21 evaluable patients, 8 patients (38.1%) achieved pCR or near-pCR, while 13 (61.9%) were classified as non-pCR. Patient demographics and tumor characteristics can be found in Table 1.

**Table 1:** Patient demographics, tumor characteristics, and preoperative radiotherapy details

Characteristic	Included cases (*N* = 23)
Age at diagnosis	
Mean (SD)	59.8 (13.3)
Median [min, max]	60 [41–85]
Sex	
Female	11 (47.8%)
Male	12 (52.2%)
Preoperative radiation therapy information	
Total dose (Gy)	
Median [min, max]	45.0 [33.6—50.0]
Dose per fraction (Gy)	
Median [min, max]	2.0 [2.0—3.0]
Fractions (*n*)	21 [14–25]
Most common treatment regimens	
50 Gy/25 fx	11 (47.8%)
36 Gy/18 fx	5 (21.7%)
42 Gy/14 fx	4 (17.4%)
Tumor location	
Upper leg	12 (52.2%)
Lower leg	6 (26.1%)
Trunk wall and pelvis	4 (17.4%)
Upper arm	1 (4.3%)
Histologic subtypes	
Myxoid liposarcoma	5 (21.7%)
Undifferentiated pleomorphic sarcoma	5 (21.7%)
Spindle cell sarcoma	3 (13%)
Undifferentiated sarcoma	3 (13%)
Myxofibrosarcoma	2 (8.7%)
Biphasic synovial sarcoma	2 (8.7%)
Liposarcoma	1 (4.3%)
Leiomyosarcoma	1 (4.3%)
Pleomorphic and spindle cell sarcoma	1 (4.3%)
Depth	
Superficial	6 (26.1%)
Superficial + deep	8 (34.8%)
Deep	9 (39.1%)
EORTC-STBSG Response Score	21 (100%)
A + B + C	8 (38.1%)
D + E	13 (61.9%)

*SD* standard deviation, *Gy* Gray, *fx* fractions, *EORTC-STBSG* European Organisation for Research and Treatment of Cancer—Soft Tissue and Bone Sarcoma Group

### Image characteristics

All images were acquired using Philips Medical Systems (Best, the Netherlands) MRI machines. ADC maps were calculated using b = 200 and b = 800 s/mm^2^ acquisitions. The follow-up MRI was acquired at a median of 13.1 weeks (92 days) after the baseline scan (range 7.3–31.0 weeks). Protocols were optimized for field strength and anatomy, with 3 T scans generally providing higher resolutions than 1.5 T acquisitions:

- 1.5 T (*n* = 12): Mean TR/TE was 4872/74.5 ms, with an average acquired resolution of 1.9 × 1.9 × 6.8 mm.
- 3 T (*n* = 30): Mean TR/TE was 3432/69.4 ms, with higher spatial resolution (1.6 × 1.6 × 5.1 mm) utilized to leverage the increased signal-to-noise ratio.

Field strength varied between baseline and follow-up in 10 patients (47.6%), reflecting clinical workflows where 1.5 T and 3 T systems may be used interchangeably. This introduced minor geometric variations (mean intra-patient difference: 1.0 mm in slice thickness and 0.19 mm for in-plane voxel resolution). Detailed acquisition parameters and consistency metrics are provided in Supplementary Material S1.

### ADC map quality and interobserver agreement

The overall diagnostic quality of the ADC maps was high. At baseline, 19/23 (82.6%) scans were rated as favorable (RIQUAL A or B) by at least two readers, and this proportion increased to 20/23 (87.0%) at follow-up. Conversely, lower-quality scores were infrequent: only 4/23 (17.4%) at baseline and 3/23 (13.0%) at follow-up were judged as C or D by at least two readers. Importantly, only a single examination across all timepoints received a strictly non-diagnostic 'D' rating from two readers simultaneously.

Despite the high proportion of diagnostically acceptable scans, overall interobserver agreement of the four RIQUAL categories separately was near-zero (Fleiss’ kappa = 0.04, *p* = 0.55). On the dichotomized scale, agreement improved somewhat (Fleiss’ kappa = 0.25, *p* < 0.05).

### ADC measurements

Assessment of the mean tumor ADC was feasible in all 23 patients for all readers. Reader 1 defined all sub-regional ROIs, but reader 2 deemed segmentation infeasible for three baseline ROIs (two high, one low) and three low-ADC ROIs at follow-up. Reader 3 could not define the high-ADC SaB ROI in one case.

#### Reproducibility

**Fig. 2:**
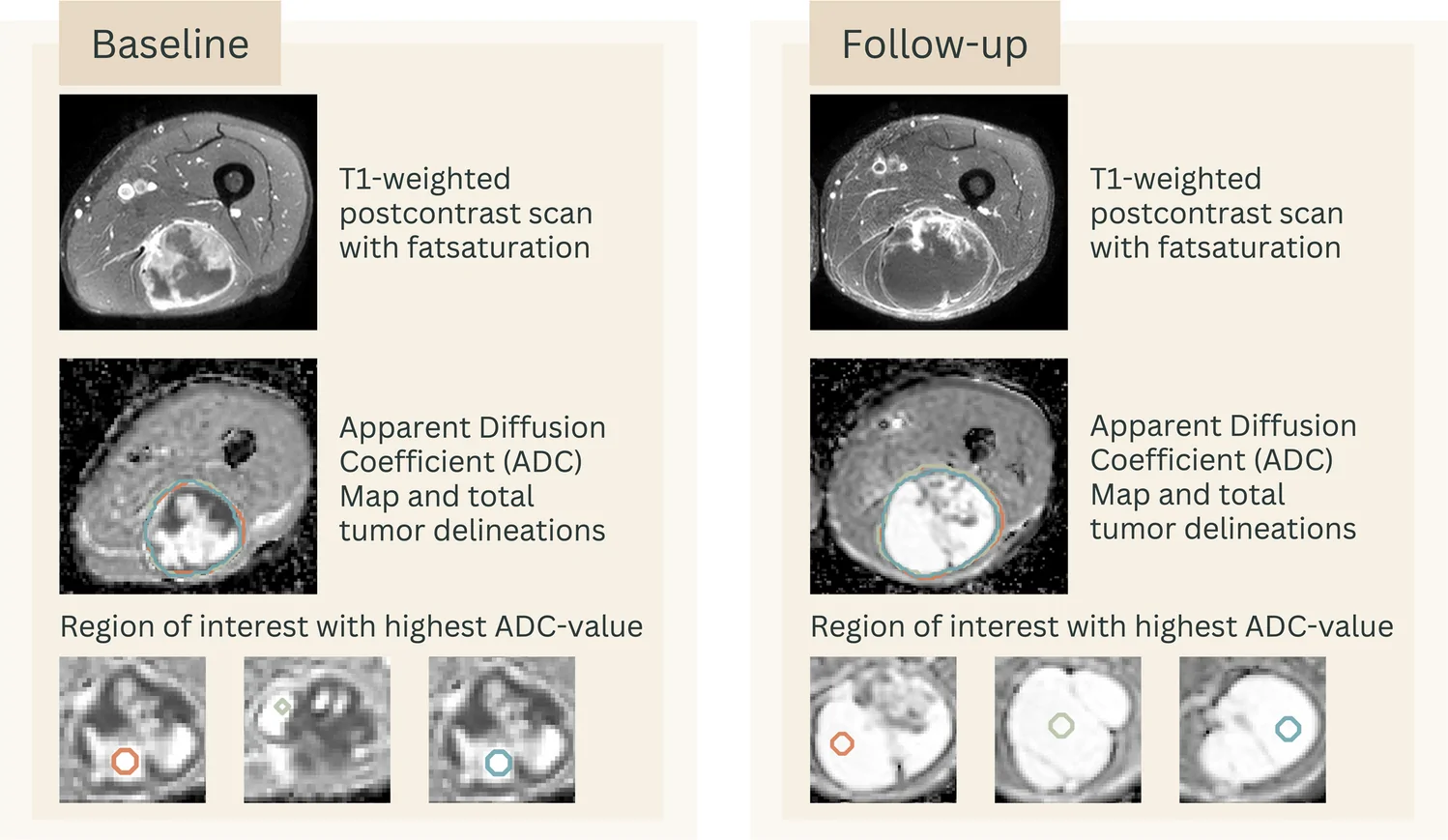
Example case demonstrating inter-reader variability in tumor delineation and high-ADC region of interest placement. Baseline (Left) and follow-up (Right) assessments of an undifferentiated pleomorphic sarcoma. The top row shows the contrast-enhanced T1-weighted fat-saturated anatomical reference images. The middle row displays the corresponding ADC maps with total tumor segmentations by three independent readers (Reader 1: Terracotta, Reader 2: Green, Reader 3: Blue). The bottom row provides zoomed-in ADC maps illustrating example placements of the "High ADC" 2D region of interest by each individual reader at both time points

Single timepoint measurements demonstrated high reproducibility (Table 2). At baseline, agreement was strongest for mean tumor ADC (ICC = 0.95), followed by low- and high-ADC ROIs (ICC = 0.89 and 0.85, respectively). At follow-up mean tumor ADC also showed highest ICC (ICC = 0.91). Sub-regional ROIs maintained decent reproducibility with ICCs of 0.82 and 0.89, though overall agreement was marginally lower when compared with baseline. Figure 2 shows an example case with total tumor delineations and high-ADC ROIs from all three readers.

**Table 2:** Inter-reader reproducibility of ADC measurements at baseline and follow-up

Timepoint	ROI	*N* pairs	ICC (95% CI)
Baseline	Mean Tumor	23	0.95 (0.90–0.99)
	Low-ADC ROI	22	0.89 (0.53–0.97)
	High-ADC ROI	21	0.85 (0.74–0.92)
Follow-up	Mean Tumor	23	0.91 (0.81–0.98)
	Low-ADC ROI	20	0.82 (0.52–0.96)
	High-ADC ROI	23	0.89 (0.78–0.96)
	Low-ADC SaB	22	0.82 (0.65–0.93)
	High-ADC SaB	20	0.66 (0.37–0.86)
Delta (∆)	Mean tumor ∆	23	0.79 (0.45–0.93)
	Low-ADC ROI ∆	20	0.06 (0.04–0.60)
	High-ADC ROI ∆	21	0.63 (0.26–0.72)
	Low-ADC SaB ∆	22	0.07 (0.05–0.73)
	High-ADC SaB ∆	20	0.53 (0.34–0.81)

*ICC* intraclass correlation coefficients, *ROI* region of interest, *SaB* same as baseline

Intraclass correlation coefficients are reported with 95% Confidence Intervals. *N* represents the number of patients for whom all three radiologists successfully defined the region of interest

Reproducibility declined when looking at the SaB ROIs. SaB high ADC ROIs had an ICC of 0.66, whereas SaB ROIs for low ADC regions demonstrated better agreement (ICC = 0.82). This suggests that ROI reproducibility decreases when regions are reused across timepoints rather than redefined based on current imaging characteristics, indicating that static ROI placement may not adequately capture dynamic spatial changes within the tumor during treatment.

Inter-reader agreement for changes over time (∆ADC) was substantially lower than for single-timepoint measurements. Agreement was highest for the mean tumor ∆ADC (ICC = 0.79). For sub-regional analysis, ∆ADC from high-ADC regions showed fair agreement (ICC = 0.63), whereas low-ADC regions were not reproducible (ICC = 0.06). A similar pattern was observed for SaB-based measurements with higher agreement in high-ADC regions (ICC = 0.53) compared to low-ADC regions (ICC = 0.07), highlighting the limited robustness of sub-regional ∆ADC metrics across readers.

#### Overall predictive value of ADC measurements

**Fig. 3:**
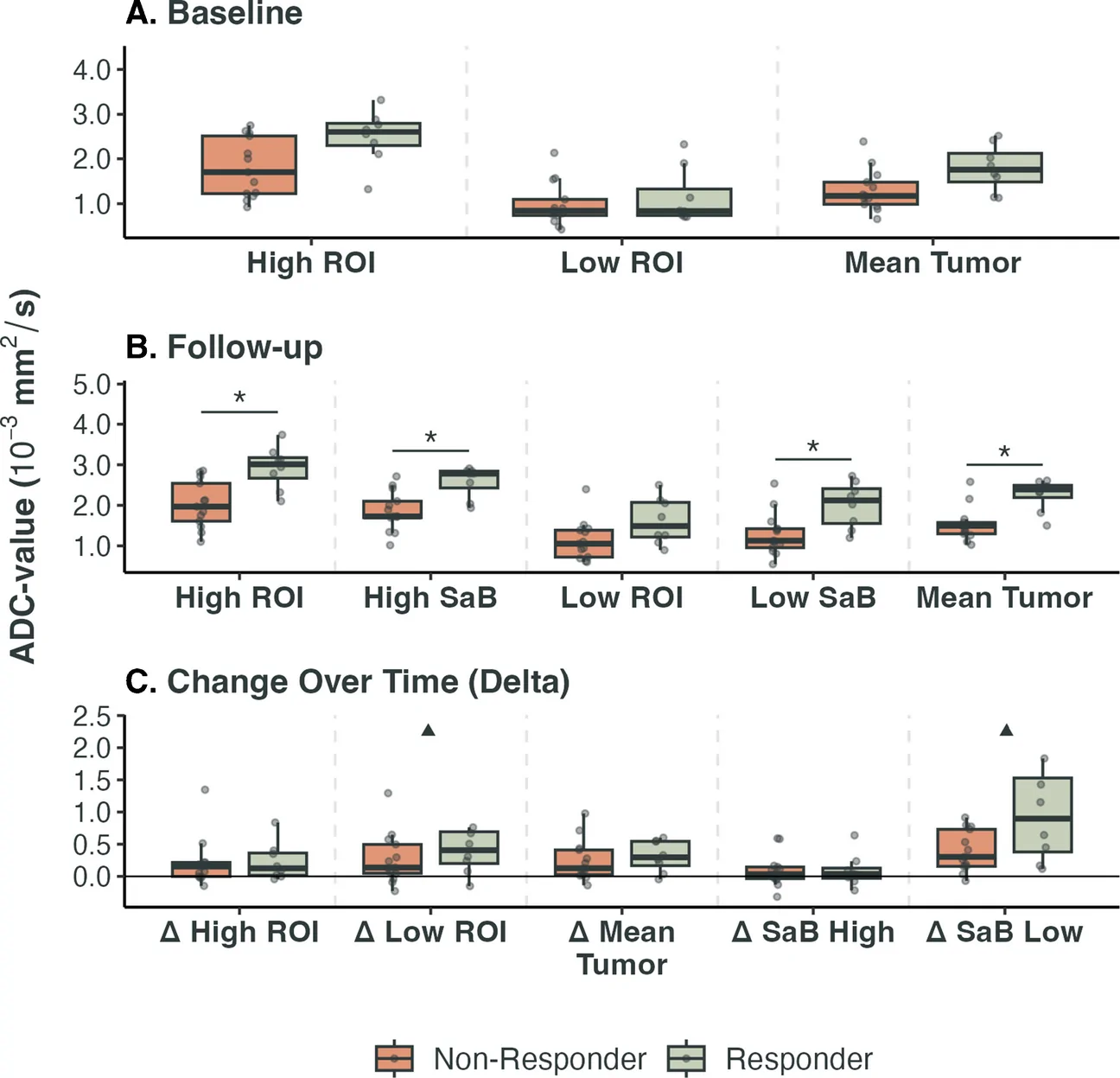
Boxplots of ADC values for different regions of interest. Results are stratified by responders vs. non-responders. **a** Baseline ADC intensities across different Regions of Interest (ROI). **b** Follow-up ADC intensities showing significant stratification between groups. **c** Change in ADC values (Delta) from baseline to follow-up. Boxplots represent the median (central line) and interquartile range (IQR, box boundaries). Individual patient data points are overlaid as jittered circles. Significant differences between non-responders (orange) and responders (green) are marked (*), indicating an FDR-adjusted *p* < 0.05. To maintain visual scale, extreme outliers exceeding the y-axis limits are indicated by triangular markers (⏶) at the plot border

**Fig. 4:**
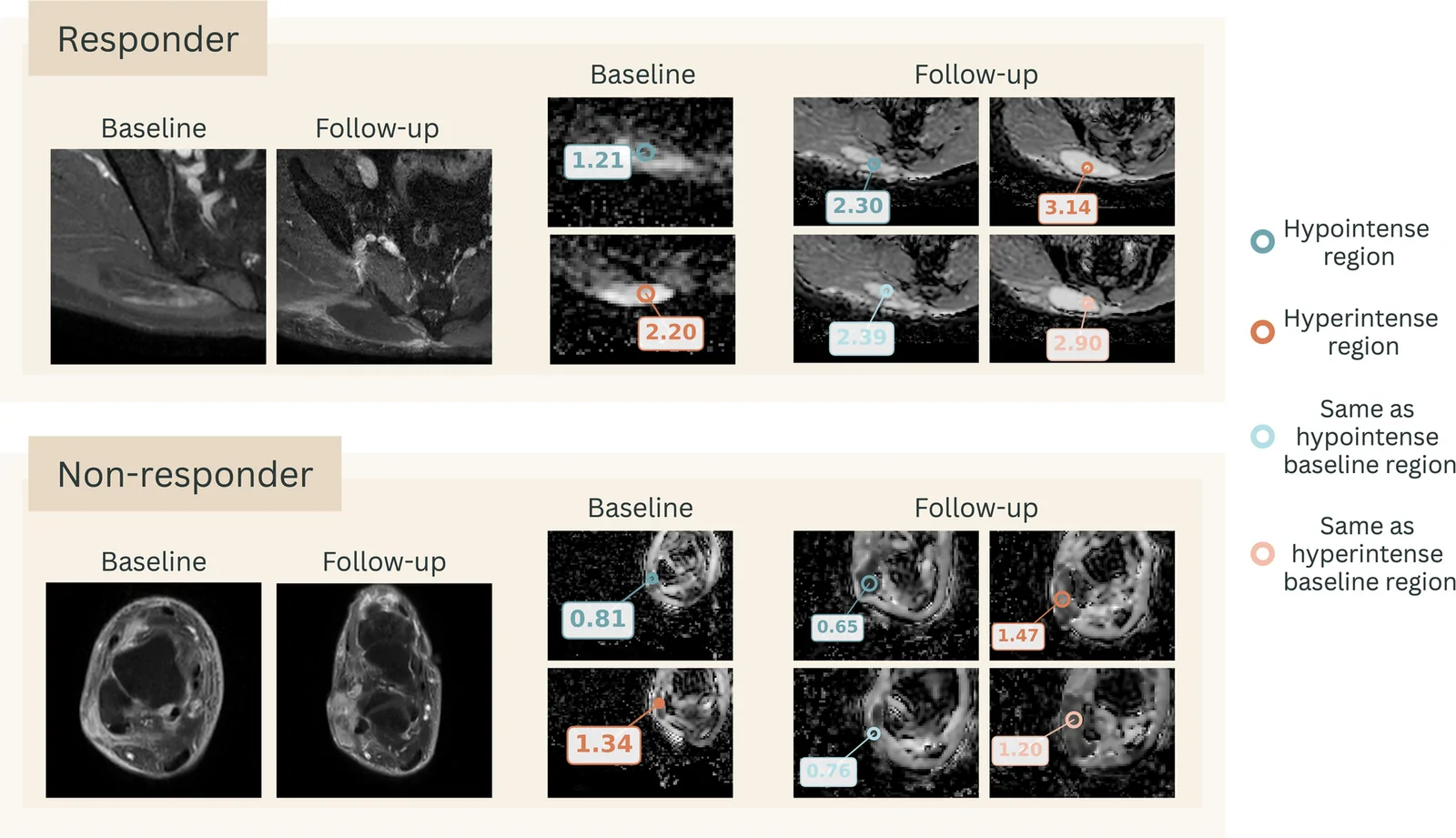
Representative responder and non-responder illustrating high- and low-ADC ROI placement at baseline and follow-up. Top: a responder (myxoid liposarcoma; 1% viable tumor cells). Bottom: a non-responder (undifferentiated sarcoma, NOS; 60% viable tumor cells). Leftmost panels show the T1-weighted post-contrast fat-saturated reference; adjacent panels show ADC maps with the hyperintense (high-ADC) and hypointense (low-ADC) regions of interest, plus the "same as baseline" regions at follow-up. ADC values (× 10⁻^3^ mm^2^/s) are annotated on each ROI. All ADC maps use identical windowing. ROIs are from a single representative reader; inter-reader variability is shown in Fig. 2 and Table 2

Differences between responders and non-responders for the various ADC-metrics are visualized in Fig. 3. Overall, responders showed higher ADC values across all metrics. However, only a subset of follow-up measurements significantly separated responders from non-responders. Mean tumor ADC was higher in responders (median 2.39 × 10⁻^3^ mm^2^/s, IQR: 2.19—2.49) compared to non-responders (median 1.50 × 10⁻^3^ mm^2^/s, IQR: 1.30—1.58; *p* = 0.02). Similarly, high-ADC ROIs showed higher values in responders (2.93 vs. 2.02 × 10⁻^3^ mm^2^/s, *p* = 0.02). SaB-based measurements demonstrated a similar pattern, with higher values in responders for both high-ADC (median 2.78 vs. 1.73 × 10⁻^3^ mm^2^/s) and low-ADC regions (mean 2.01 vs. 1.29 × 10⁻^3^ mm^2^/s). Representative responder and non-responder cases illustrating these differences in the high- and low-ADC ROIs are shown in Fig. 4. In contrast, the change in whole-tumor volume did not differ significantly between responders (median + 15.1%, IQR − 50.4 to + 120.5) and non-responders (median + 36.7%, IQR − 9.9 to + 44.1; *p* = 0.74). Longitudinal trajectories of individual patient ADC values are visualized in Supplemental Material S2.

#### Predictive power of individual measurements

In an exploratory analysis, we examined the relationship between imaging features found to be significant after multiple comparison correction and pathological response. These were mean tumor ADC and sub-regional high- and low-ADC metrics at follow-up.

Univariable logistic regression demonstrated that higher values for the high-ADC region at follow-up were associated with markedly increased odds of response (OR = 20.3, 95% CI: 2.68–551, *p* = 0.02). The same was observed for mean tumor ADC at follow-up (OR = 30.3, 95% CI: 3.35–681, *p* = 0.009), SaB-derived low-ROI (OR = 9.36, 95% CI: 1.71–98.2, *p* = 0.024) and SaB-derived high-ROI (OR = 33.1, 95% CI: 3.2–1058, *p* = 0.014). Confidence intervals were wide across all models, reflecting variability and limited sample size.

**Fig. 5:**
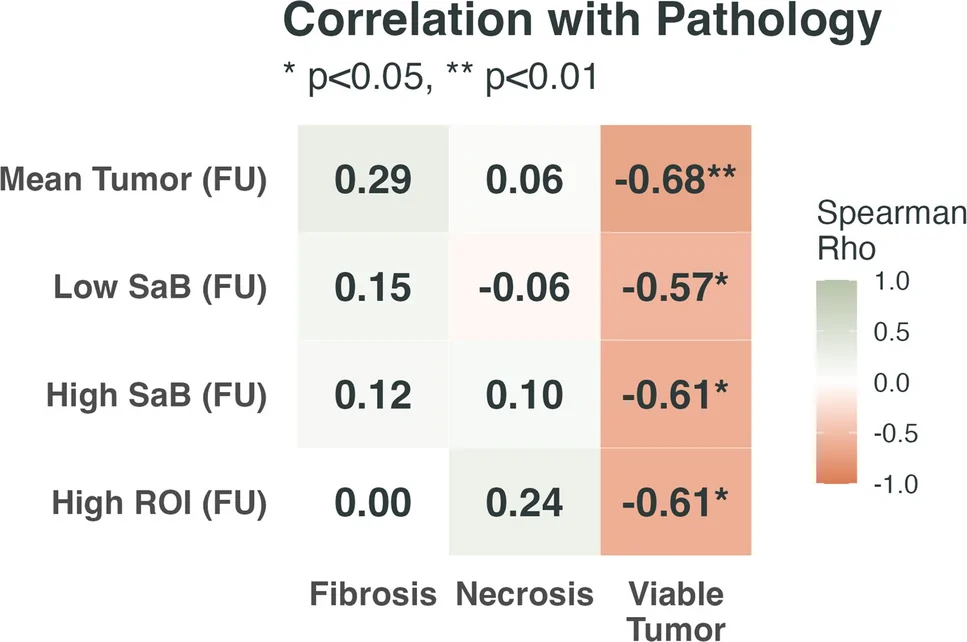
Correlation heatmap of ADC metrics and pathological markers. The heatmap displays Spearman’s rank correlation coefficients between significant follow-up ADC metrics and histological findings. Significance indicated by * *p* < 0.05, ** *p* < 0.01 (FDR-corrected)

Consistent with these findings, Spearman rank correlation demonstrated an inverse relationship between follow-up ADC values and the proportion of viable tumor cells (Fig. 5), indicating that lower ADC values are associated with a higher proportion of viable tumor tissue.

## Discussion

In this study, we explored the use of quantitative ADC mapping as a non-invasive biomarker for treatment response in patients with STS treated with nRT. Our primary finding is that absolute ADC values obtained at follow-up seem to be predictive of pathological response. In this cohort, responders consistently demonstrated higher post-treatment ADC values compared to non-responders, with a clear inverse relationship between ADC and the proportion of viable tumor cells. From a radiological perspective, this supports the use of follow-up ADC as a practical imaging surrogate for residual tumor viability.

This relationship is biologically plausible. Effective therapy induces cell death and membrane disruption, reducing cellular density and increasing extracellular water mobility. As a result, diffusivity increases which is reflected in higher ADC values. In contrast, viable tumor tissue remains highly cellular, restricting diffusion and resulting in lower ADC values. Our results align with previous reports in STS applying EORTC criteria, which demonstrated that responders typically exhibit significant post-treatment elevations in mean ADC [3, 11, 23, 31, 32].

For radiologists, however, predictive performance alone is insufficient. Quantitative metrics must also be reproducible. We found a critical discrepancy between the reliability of absolute measurements versus longitudinal change metrics. Single-timepoint ADC measurements proved excellent reproducibility across readers, with ICCs consistently exceeding 0.9 for TTV at both baseline and follow-up. These findings confirm that absolute ADC values can be reliably measured in STS when standardized ROI definitions are applied, consistent with prior work on whole-volume segmentation in retroperitoneal sarcomas [21].

In contrast, reproducibility declined substantially when assessing longitudinal changes (∆ADC), particularly for subregional analyses. This has important practical implications. While delta metrics are intuitively appealing for treatment monitoring, their reliability appears limited in this setting. Sub-regional approaches such as SaB ROIs were especially vulnerable, likely due to the difficulty of consistently re-identifying the same anatomical regions after treatment-induced morphological changes. Whole-tumor ADC appears more robust, likely because it averages out intratumoral heterogeneity, although even tumor-wide ∆ADC showed variability. These findings suggest that radiologists should be cautious in relying on sub-regional or delta-based ADC metrics in clinical decision-making. Emerging approaches such as habitat-based analysis may offer a more stable alternative for capturing tumor heterogeneity [22].

Several factors likely contribute to the reduced reproducibility of longitudinal metrics. Biologically, the heterogeneity of STS plays a significant role. These tumors contain variable components of necrosis, hemorrhage, and fluctuating cellularity that evolve unevenly during therapy. Consequently, selected sub-regions may reflect local fluctuations rather than global therapeutic effect [17, 33]. From a technical perspective, ∆ADC is inherently sensitive to error propagation. Combining two independent measurements amplifies the noise inherent in each. Variations in acquisition parameters and image quality between baseline and follow-up can introduce further error. Notably, in our cohort baseline and follow-up scans were acquired at different field-strengths in nearly half of the cases, which likely contributed to measurement variability [34, 35]. This aligns with prior multicenter studies demonstrating sub-regional ∆ADC are particularly susceptible to noise and variability [36, 37].

Despite these limitations, it is important to emphasize that our study reflects real-world clinical practice. Soft tissue sarcoma is a rare and heterogeneous disease for which imaging is often performed across multiple institutions with varying protocols, for example a diagnostic scan in a general hospital and follow-up in a specialized centre. Recent data show that even guideline-recommended sequences such as DWI are frequently absent in referral imaging, with 78.1% of patients missing the DWI sequences [38]. In this context, the fact that absolute ADC measurements were reproducible in our cohort despite non-standardized acquisition parameters is a key strength. It suggests that robust ADC metrics can be applied in realistic clinical settings, where perfect standardization is often not achievable. This is particularly relevant given that current response assessment frameworks, including RECIST and Choi, are also not tailored to specific histological subtypes.

Regarding the reference standard, the optimal definition of histopathologic response in STS remains debated. We focused on the percentage of viable tumor cells in accordance with EORTC recommendations, as this parameter is currently considered the most reliable indicator of treatment effect [11, 39–41]. In contrast, necrosis and fibrosis are more controversial, with conflicting evidence regarding their association with survival [9, 42]. In our cohort, higher follow-up ADC values were associated with increased odds of response as defined by viable tumor content, although confidence intervals were wide, reflecting the limited sample size.

This study has limitations. The cohort was relatively small and histologically heterogeneous, which may limit generalizability and precludes subtype-specific analysis. However, this reflects the clinical reality of STS, where large homogeneous datasets are difficult to obtain. Future studies should aim to validate these findings in larger, ideally multicenter cohorts. In addition, histopathologic response was assessed from representative tissue sections rather than whole-tumor spatial mapping, so the subregional ROIs delineated on imaging may not correspond to the exact regions sampled histopathologically. Imaging protocols were also not fully standardized across all examinations, which may have introduced variability in ADC measurements. Nevertheless, the high reproducibility of single-timepoint whole-tumor ADC supports its feasibility as a quantitative imaging biomarker in multicenter settings, provided that acquisition parameters are reasonably harmonized. These findings further underscore the importance of routinely including diffusion-weighted imaging in STS MRI protocols, as consistent acquisition will be essential to enable robust quantitative analysis and facilitate future research in treatment response assessment.

Beyond standardized acquisition, the main challenge that remains is the reliability of subregional and longitudinal metrics. Both depend on manual ROI placement, which was the main source of variability in our cohort. Removing manual selection would therefore be the most direct route to improvement: AI-based (semi-)automated segmentation reduces variability in tumor delineation [43], while data-driven partitioning of the tumor into subvolumes, such as habitat analysis [22], could replace manually ROI placement.

In summary, quantitative ADC measurements in STS demonstrated excellent reproducibility when based on TTV in our cohort, whereas longitudinal differences in ADC showed limited reliability. Follow-up ADC correlated with pathological response, reflecting its potential as a surrogate marker of tumor viability after neoadjuvant radiotherapy. These findings support the potential of follow-up ADC as an imaging biomarker of treatment response in STS. However, they also highlight the limitations of longitudinal and sub-regional approaches and emphasize the importance of standardized diffusion-weighted imaging protocols. Broader and more consistent inclusion of DWI in routine sarcoma MRI protocols will be essential to enable reproducible quantitative imaging and facilitate future multicenter validation studies across histologic subtypes.

## Supplementary information

Below is the link to the electronic supplementary material.

ESM 1 (DOCX 451 KB)

## Data Availability

The data supporting this study consist of patient medical imaging data and are not publicly available due to privacy restrictions. Access requests may be directed to the corresponding author.
