# Supplementary material for: Apparent diffusion coefficient metrics for treatment response assessment in soft tissue sarcoma: Reproducibility and prognostic value of whole-tumor and sub-regional analysis
Source: Skeletal Radiol. 2026 Jul 30;55(11):2845–55. doi: 10.1007/s00256-026-05318-9 (PMC13574881; doi:10.1007/s00256-026-05318-9)
Supplement: Supplementary file 1 [file 256_2026_5318_MOESM1_ESM.docx]

# Supplements

**Table S1. Detailed MRI Acquisition Parameters and Longitudinal Consistency**

| **Parameter** | **1.5 T Scans (n=12)** | **3.0 T Scans (n=30)** | **Mean Intra-patient Difference † (n=21)** |
| --- | --- | --- | --- |
| **Repetition Time (TR), ms** | 4872 (2381–8004) | 3432 (1200–5141) | — ‡ |
| **Echo Time (TE), ms** | 74.5 ± 5.8 | 69.4 ± 10.3 | 7.6 ± 7.1 |
| **Field of View (FOV), mm** | 327 ± 72 | 324 ± 118 | 84.5 ± 71.8 |
| **In-plane Resolution, mm** | 1.9 ± 0.1 | 1.6 ± 0.1 | 0.19 ± 0.15 |
| **Slice Thickness, mm** | 6.8 ± 0.6 | 5.1 ± 0.5 | 1.0 ± 1.1 |

Overview of acquisition parameters. Data are presented as mean ± standard deviation or mean (range).

† Mean Intra-patient Difference**:** Calculated as the mean absolute difference between baseline and follow-up values.

‡ TR: Differences in TR were not quantified longitudinally as they are primarily driven by physiological gating rather than protocol inconsistency.

**Figure S2. Longitudinal trajectories of individual patient ADC values**
